# Real-Time Monitoring of Antibiotics in the Critically Ill Using Biosensors

**DOI:** 10.3390/antibiotics12101478

**Published:** 2023-09-22

**Authors:** Ruvimbo Dephine Mishi, Michael Andrew Stokes, Craig Anthony Campbell, Kevin William Plaxco, Sophie Lena Stocker

**Affiliations:** 1 Department of Human Biology, Division of Cell Biology, University of Cape Town, Cape Town 7925, South Africa; 2 Paediatric Critical Care Unit, Department of Pharmacy, The Children’s Hospital at Westmead, Sydney, NSW 2031, Australia; 3 NSW Health Pathology, Department of Chemical Pathology, Prince of Wales Hospital, Sydney, NSW 2031, Australia; 4 Department of Chemistry and Biochemistry, University of California Santa Barbara, Santa Barbara, CA 93106, USA; 5 Center for Bioengineering, University of California Santa Barbara, Santa Barbara, CA 93106, USA; 6 Biomolecular Sciences and Engineering, University of California, Santa Barbara, CA 93106, USA; 7 School of Pharmacy, Faculty of Medicine and Health, The University of Sydney, Sydney, NSW 2006, Australia; 8 Department of Clinical Pharmacology and Toxicology, St. Vincent’s Hospital, Sydney, NSW 2010, Australia; 9 Sydney Institute for Infectious Diseases, University of Sydney, Sydney, NSW 2006, Australia

**Keywords:** biosensor, therapeutic drug monitoring, antibiotic, intensive care unit, critically ill, aptamers

## Abstract

By ensuring optimal dosing, therapeutic drug monitoring (TDM) improves outcomes in critically ill patients by maximizing effectiveness while minimizing toxicity. Current methods for measuring plasma drug concentrations, however, can be challenging, time-consuming, and slow to return an answer, limiting the extent to which TDM is used to optimize drug exposure. A potentially promising solution to this dilemma is provided by biosensors, molecular sensing devices that employ biorecognition elements to recognize and quantify their target molecules rapidly and in a single step. This paper reviews the current state of the art for biosensors regarding their application to TDM of antibiotics in the critically ill, both as ex vivo point-of-care devices supporting single timepoint measurements and in vivo devices supporting continuous real-time monitoring in situ in the body. This paper also discusses the clinical development of biosensors for TDM, including regulatory challenges and the need for standardized performance evaluation. We conclude by arguing that, through precise and real-time monitoring of antibiotics, the application of biosensors in TDM holds great promise for enhancing the optimization of drug exposure in critically ill patients, offering the potential for improved outcomes.

## 1. Therapeutic Drug Monitoring in the Critically Ill Is Challenging

The safe and effective dosing of drugs in critically ill patients remains a major clinical challenge, particularly for antibiotics. The problem is two-fold. First, the therapeutic indices of many critical antibiotics are narrow. For example, vancomycin has a desired therapeutic target area under the curve to minimum inhibitory concentration ratio of 400–600. Second, critically ill patients, the population for whom the margin for clinical error is the smallest, often undergo rapid, clinically significant changes in physiology that, in turn, often lead to clinically significant variations in pharmacokinetics and pharmacodynamics. Endothelial dysfunction and intravenous fluid loading, for instance, can significantly increase the volume of distribution of hydrophilic antimicrobials, altering pharmacokinetics. Similarly, critical illness is often associated with altered renal clearance, also further contributing to intra- and inter-patient pharmacokinetic variability [1]. Consequently, using standard antibiotic doses may not be appropriate.

In response to the difficulty in accurately dosing antibiotics, therapeutic drug monitoring (TDM) has become the standard of care for many antibiotics, including vancomycin, aminoglycosides, and linezolid and increasingly being considered for beta lactam antibiotics [2]. With this approach, knowledge of a drug’s plasma concentration is used to inform future drug dosing. That is, if the concentration is below the desired concentration, the dose is increased and vice versa. TDM currently requires the laboratory analysis of drawn blood to determine the drug concentration. And given that drug concentrations fluctuate, this sample needs to be obtained at a specific time point in the dosing interval (usually a trough concentration) to enable interpretation. Together with the cumbersome nature of blood draws and benchtop analysis, this requirement for well-timed sample collection contributes significantly to the difficulty of performing TDM in busy clinical environments [3,4].

Given the burdens associated with obtaining blood samples at precise times, state of the art has more recently moved to model-informed precision dosing (MIPD), an approach that enables TDM to be performed using drug concentrations obtained at any time within the dosing interval. MIPD uses mathematical models combined with individual characteristics to describe the pharmacokinetics of a drug in a patient. In doing so, MIPD makes predictions of drug exposure (area under the curve values or trough concentrations, as relevant) and recommends dose regimens to achieve pharmacokinetic or pharmacokinetic/pharmacodynamic targets. Despite the clear advantages of MIPD, however, the approach is not without limitations. The mathematical models commonly used in MIPD software, for example, do not always capture the range of pharmacokinetics and pharmacodynamics seen across heterogeneous critically ill populations. Numerous studies have highlighted the difficulty that such models have in accurately predicting the pharmacokinetics in critically ill patients for multiple antibiotics, including vancomycin [5,6], amikacin [4], gentamicin, tobramycin [7], meropenem [8], ciprofloxacin [9], cefotaxime [10], and polymyxin [11]. This problem is exacerbated in critically ill patient groups, such as pediatrics or those undergoing renal replacement therapies, whose pharmacokinetic profiles are distinct from those of more typical patients. And while the accuracy of MIPD-informed dose recommendations can be improved through entry of additional drug concentrations into the modelling software, this requires more frequent blood sampling, negating one of the key benefits of MIPD [12].

## 2. Current Analytical Approaches to Determining Drug Concentrations

The ideal approach to TDM in the critical care setting will be minimally invasive, will provide results immediately (i.e., in real-time), and will allow for easy interpretation of concentrations at the bedside. Current approaches for the measurement of drug concentrations, however, which include immunoassays and liquid chromatography–tandem mass spectrometry (LC-MS/MS), rely on the ex vivo analysis of collected blood samples in the laboratory, precluding their use in performing continuous real-time drug monitoring. In contrast, examples of reagent-less, rapidly reversible analytical approaches have recently been described in the literature that support point-of-care testing and continuous real-time molecular measurements in situ in the body. Here we review these technologies considering their potential application to TDM.

## 3. Application of Biosensors for TDM

Over time, natural selection has propagated workable solutions for biochemical processes that require molecular identification, mastering the ability to respond quantitatively to specific molecular cues continuously and in real time in the complex environments found within the body. Given this, biosensors, molecular measurement technologies that employ biorecognition elements to recognize their targets, seem a likely potential solution to the problem of monitoring drugs in the body in real time. Here we explore the potential use of biosensors in antibiotic TDM.

**Figure 1:**
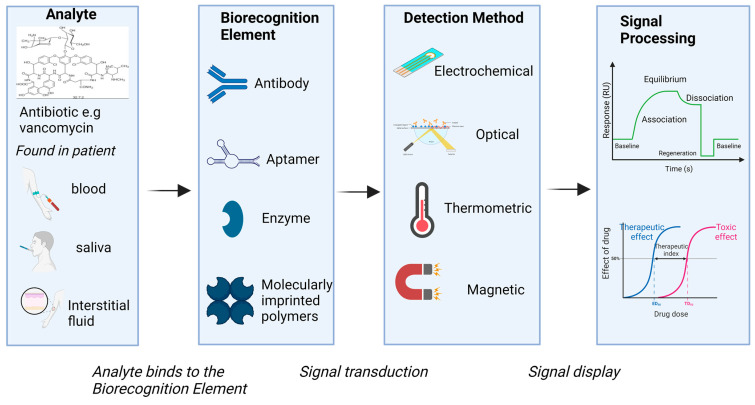
Overview of biosensors including their biorecognition elements, detection method, and signal processing.

Biosensors are classified according to their transducing element and can be divided into electrochemical-, optical-, magnetic-, and thermometric-based biosensors (Table 1 and Figure 1). Biorecognition elements employed in biosensors include naturally occurring biomolecules (e.g., antibodies and enzymes), artificially generated biomolecules (e.g., aptamers), and even synthetic biomimetic molecules (e.g., molecularly imprinted polymers) [13,14] (Table 2). Due to the broad generalizability of antibodies, immunochemical approaches dominate in the laboratory. Antibody-based biosensors [15,16], however, have seen little penetration to quantitative point-of-care tests, much less to in vivo applications. This is because antibodies do not “respond” to target binding, i.e., they do not emit photons or electrons, do not produce some new, easily detectable molecule, and do not even change their shape upon binding. Given this problem of “transducing” an antibody binding event into a measurable output, antibodies are typically employed in multistep assays rather than quantitative single-step devices. This renders them inconvenient for use in point-of-care devices and entirely unable to support continuous real-time monitoring in vivo.

In contrast to antibodies, some enzymes respond to target binding by converting their target molecules into easily detectable products. Enzymatic biosensors, which rely on this ability, were the first biosensors to monitor specific molecules in the body in real time. The most prominent example of these, the continuous glucose monitor [21,22] worn by tens of millions of patients, typically employs the enzyme glucose oxidase which, in the presence of glucose and oxygen, produces easily detectable peroxide (or electrons) in a single continuous step. Other similar examples include biosensors supporting real-time lactate [23] and glutamate [24] monitoring, which have been used for research but not yet transitioned into the clinic. While these examples all monitor metabolites of endogenous compounds, the first enzymatic biosensor supporting human in vivo drug monitoring was recently reported. This biosensor uses β-lactamase to monitor the concentration of phenoxymethylpenicillin via the enzyme’s production of protons, which are subsequently detected via a pH electrode [25]. The success of the continuous glucose monitors notwithstanding, the reliance of this and other enzymatic biosensors [26] on the chemical transformation of their target molecule limits the adaptation of such biosensors to new targets as enzymes with the necessary properties simply do not exist for most drugs.

Motivated by the limited scope of enzymatic biosensors, a new biosensing approach has recently been developed that embodies both the generality of antibody-based detection (i.e., similarly, it does not require the chemical transformation of the target) and the single-step real-time monitoring abilities of enzymatic sensors. This new class of devices employs aptamers, synthetic oligonucleotides generated by an in vivo evolutionary process, as its biorecognition element. In these biosensors [27,28,29,30,31,32], target binding changes the shape of an electrode-bound aptamer, altering the rate of electron transfer from an attached redox reporter [33] and producing an easily measurable signal change when the biosensor is interrogated electrochemically. Because this conformation-linked signal mechanism mimics the signaling of naturally occurring receptors (such as G-protein-coupled receptors), it is selective enough to perform well in bodily fluids [34]. And because both the aptamer conformational change, and target binding and unbinding are rapid and rapidly reversible, sensors in this class support high frequency (typically every few seconds) real-time monitoring.

## 4. Application of Biosensors to Individualize Antibiotic Use in the Critically Ill

Biosensor-enabled antibiotic TDM in the critically ill can be conducted using either single timepoint ex vivo measurements (analogous to the finger prick glucose test or finger prick sample blood gas monitoring) or continuous monitoring (analogous to the wearable continuous glucose monitor). Both approaches mitigate the slow turnaround times which have traditionally complicated the interpretation of drug concentrations in critically ill patients in intensive care. Table 3 provides a summary of the state of the art in the development of biosensors suitable for monitoring antibiotic concentrations.

### 4.1. Point of Care Devices for Single Timepoint Measurements

One approach to implementing biosensors for TDM would be to use them as point-of-care devices for rapidly performing ex vivo measurements. Recently a non-antibody point-of-care biosensor was shown to detect the concentration of piperacillin/tazobactam in whole blood, plasma, urine, and saliva of pigs [36] (Table 3). A modified version of the biosensor was able to detect piperacillin/tazobactam and meropenem simultaneously in the pig plasma. Whilst the piperacillin/tazobactam concentrations obtained from the biosensor were similar to those obtained by high-performance liquid chromatography (HPLC), the anesthetic drug propofol was shown to produce significant positive interference in the biosensor readings, resulting in the overestimation of piperacillin concentrations. This lack of specificity was likely due to the employment of penicillin-binding protein 3 as the biomolecular recognition element in this particular model and should not be viewed as representative of biosensor specificity in general.

### 4.2. Continuous Real-Time Measurements

A potentially higher value application of biosensors for antibiotic TDM in intensive care patients would be as wearable devices supporting continuous real-time measurements. Continuous real-time monitoring would be particularly useful for antibiotics administered by continuous infusions where dose rates can be easily adjusted in response to changes in drug concentrations. In silico studies based on pharmacokinetic data of piperacillin obtained in adults with sepsis [15] and vancomycin in noncritically ill adults [16] demonstrate the potential of these closed-loop systems to assist in achieving and maintaining optimal antibiotic exposure in critically ill patients.

One approach to achieving continuous real-time monitoring using biosensors is to extract small volumes (<1 nL) of biological fluids, using hollow microneedles, and then measure drug concentrations within the lumen of the microneedles. Such microneedles are minimally invasive, penetrating the stratum corneum without reaching the nerve and blood vessels (i.e., sampling interstitial fluid). An enzymatic biosensor using this approach has been developed for the detection of vancomycin [37]. In vitro evaluation of this biosensor indicates high sensitivity and good measurement frequency (>12 measurements/h). Similarly, a single example of an electrochemical aptamer-based biosensor has been shown to work in the fluid-filled lumen of a microneedle on a human subject, with the target molecule simply diffusing into the device where it hits the sensor [38]. Further testing is required, however, to ascertain the performance (quantification, limit of detection, and specificity) of these biosensors relative to current analytical methods, particularly in the presence of polypharmacy.

Progress has also been reported in the continuous real-time monitoring of drugs using biosensors placed in situ in the body. For example, Rawson et al. have demonstrated the continuous measurement of phenoxymethylpenicillin concentrations in the interstitial fluid of humans using a biosensor that employs beta-lactamase, which liberates a detectable proton upon hydrolyzing its target [25]. The phenoxymethylpenicillin concentrations they obtained were similar to those measured by liquid chromatography–tandem mass spectrometry (LC/MS/MS) in microdialysate and blood samples concurrently collected from the study subjects. This is the most promising step forward for the clinical application of biosensors to the real-time continuous monitoring of antibiotics in humans. We suspect, however, that it may prove difficult to move this approach to clinical practice due to the challenges of monitoring target-induced changes in proton concentration in the body, where buffering agents resisting pH changes are at quite high concentration and can vary significantly from individual to individual and with health status [39].

Moving beyond enzymatic sensors, recent advances suggest that electrochemical aptamer-based sensors may prove a promising means of monitoring drugs in situ in the body. Specifically, while these have not yet reached the clinic, they have achieved notable success in animal models. Examples include the seconds-resolved measurement of plasma tobramycin, gentamycin, kanamycin, and vancomycin concentrations in situ in the veins [16,27,40,41] of live rats. Using such highly time-resolved data, time-varying changes in physiology (such as renal function) and their impact on the pharmacokinetics of antibiotics (many of which, for example, are renally cleared) can easily be seen [42]. More recently, several groups have demonstrated the real-time measurement of drug concentrations (vancomycin and tobramycin) in subcutaneous interstitial fluid [28,43], a sample matrix that can be accessed using minimally invasive microneedles. Notably, the area under the curve (AUC) of the drug concentrations in interstitial fluid, obtained using the microneedle-supported electrochemical aptamer-based sensor, correlated well with the AUC of the drug concentrations measured in blood using LC/MS/MS [28].

The availability of continuous highly time-resolved data regarding in vivo drug concentrations may require “rethinking” of how TDM is performed in the intensive care environment. Firstly, the amount of data produced with near-instant sampling by biosensors is unprecedented and may be viewed as excess to requirements by healthcare professionals who are used to targeting single-point trough concentrations. This problem will likely need to be resolved through education, pharmacy input, and clinical decision support. Given the real-time concentration information provided by in vivo biosensors, this problem could ultimately be surmounted via the use of closed-loop feedback-controlled drug delivery, which to date has been demonstrated for both vancomycin [27] and tobramycin [44] in rats. However, ensuring the longevity of the biosensor device in vivo could pose a challenge, as degradation in signal quality has been demonstrated over time and current designs have only been tested up to 12 h [45]. In intensive care, this will cover two to three dosing intervals of the most common antibiotics such as beta-lactams but may not be sufficient for assessing day-to-day trends for drugs such as tobramycin, which are commonly dosed only once daily. A perhaps more fundamental problem is that optimal therapeutic ranges are based on serum or plasma concentrations, commonly using a total (bound plus unbound) drug. Considering this, it will be crucial to determine the relationships between biosensor-measured values, likely the concentration of a free (unbound) drug in the subcutaneous interstitial fluid, and the plasma or serum values that clinical decision making is currently based on. This said, we note that drug concentrations obtained in interstitial fluid, rather than concentrations obtained in serum or plasma, may more closely reflect concentrations at the site of infection, highlighting the potential for in vivo biosensors to drive improved treatment outcomes and patient care. 

## 5. Clinical Development of Biosensors

**Figure 2:**
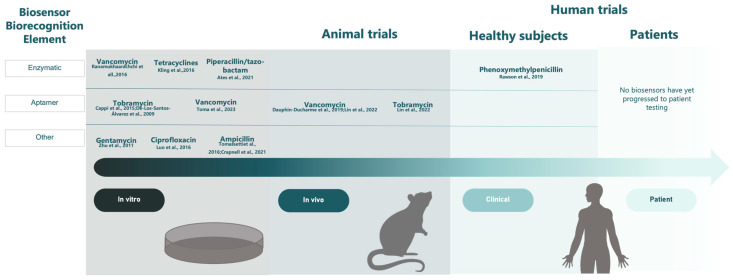
Timeline of clinical biosensors for the measurement of antibiotics. Biosensors listed as enzymatic also include sandwich assay-based biosensors [13,15,16,23,25,26,27,28,30,32,35,36,37].

Although the development of biosensors for TDM antibiotics is an active area of research, other than the glucose monitor, no real-time in vivo biosensor has been approved by regulatory agencies for clinical use. Most are still in the proof-of-concept stage with either in vitro evaluation only or in vivo testing in preclinical species (Figure 2). Currently, only the enzyme-based biosensor developed by Rawson et al. (2019) and a single example of an electrochemical aptamer-based sensor [38] have been evaluated in humans. Biosensors face several challenges to gain regulatory approval including the standardization of testing protocols, manufacturing adequate devices for clinical studies, and the constantly changing regulatory environment for medical devices [46]. The adequate accuracy, sensitivity, and specificity of biosensors in detecting analytes must also be robustly demonstrated to be in line with current performance standards for traditional analytical approaches to ensure reliability for clinical implementation. This said, it is reassuring that no significant safety concerns have arisen during animal or human testing of any biosensors, and the limit of detection (LOD) of the devices reported in studies fit within anticipated clinical ranges. It is nonetheless important to note that the stated LODs in most studies have been determined under unique laboratory conditions and may not be reproducible in real-world patients, nor can they be directly compared.

## 6. Conclusions

Biosensors have the potential to provide far more rapid and even real-time information on drug exposure, either as a timepoint or continuously, thereby fundamentally changing the way antibiotic TDM is conducted in critically ill patients. To this end, some enzymatic biosensors have been developed and evaluated, but this approach is limited in scope by the need to identify an enzyme that reacts with the targeted drug to produce an easily measurable output. Electrochemical aptamer-based biosensors, in contrast, show promise for widespread application to TDM as they are not dependent on the existence of naturally occurring biorecognition elements that produce a detectable product. As research in this space rapidly continues, the next phase will be in the validation of biosensors in patient populations where specificity issues and the longevity of the devices must be addressed. Overcoming these challenges will pave the way for integrating biosensors into routine clinical care in the intensive care unit, representing a significant leap forward in personalized medicine and improved patient care.

## Figures and Tables

**Table 1:** Types of biosensors, including their detection method, recognition element, and mechanism of functioning.

Detection Method	Recognition Element	Mechanism
Electrochemical	DNA, antibodies, membranes, and aptamers	Uses an electrode transducer that translates chemical signal into measurable electric signal, e.g., current or voltage change [17]
Optical	Enzymes, antibodies, aptamers, cells or tissue, and molecularly imprinted polymers [18]	Analyte leads to a signal through interactions with light, such as laser or LED. The signal produced correlates to the concentration of the measured analyte [18]
Thermometric	Enzymes	Measures the changes in temperature of the circulating fluid after the reaction of a substrate with immobilized enzyme [19]
Magnetic	Magnetic nanoparticles	Detect magnetic micro and nanoparticles in microfluidic channels using magnetoresistance [20]

**Table 2:** Comparison of aptamers to traditional biorecognition elements: advantages and benefits.

	Aptamers	Antibodies	Enzymes	Molecularly Imprinted Polymers
**Thermal stability**	High	Low	Low	High
**Production time**	Few months	>6 months	Several weeks	Few weeks
**Production method**	Developed using chemical modifications or SELEX	Must be obtained from animals	Fermentation	Chemical synthesis
**Production cost**	Medium	High	High	Low
**Binding Affinity**	Moderate	Moderate	High	High
**Specificity**	High	High	High	High

**Table 3:** Biosensors developed and the antibiotics measured.

Biorecognition Element	Sensor	Antibiotic	Matrix	References
Aptamer	Microneedle-based electrochemical aptamer biosensing patch (µNEAB-patch)	Vancomycin Tobramycin	Interstitial fluid	[28]
	Electrochemical aptamer based (EAB)	Vancomycin Ampicillin	Blood Saliva	[27] [29]
	Long-range surface plasmon	Vancomycin	Serum	[30]
	Surface plasmon resonance	Neomycin B Tobramycin	Solution Blood	[31,35]
	Transmission-localized surface plasmon resonance (T-LSPR)	Tobramycin	Buffer Undiluted Blood Serum	[32]
Enzyme ^a^	Electrochemical	Tetracyclines and streptogrammins	Human plasma	[26]
	Electrochemical	Piperacillin/tazobactam	Plasma Saliva Urine Whole blood	[36]
	Optical	Vancomycin	Interstitial fluid	[37]
	Microneedle-based β-lactam	Phenoxymethylpenicillin	Interstitial fluid	[25]
Antibody	Surface plasmon resonance	Ampicillin Gentamycin	Solution buffer Solution buffer	[15] [16]
Molecularly imprinted polymers	Surface plasmon resonance	Ciprofloxacin	Solution buffer	[13]

^a^ Biosensors listed as enzymatic also include sandwich assay-based biosensors.

## Data Availability

Data sharing not applicable.
